# Screening for potential novel probiotic *Levilactobacillus brevis* RAMULAB52 with antihyperglycemic property from fermented *Carica papaya* L.

**DOI:** 10.3389/fmicb.2023.1168102

**Published:** 2023-06-20

**Authors:** Navya Sreepathi, V. B. Chandana Kumari, Sujay S. Huligere, Abdel-Basit Al-Odayni, Victor Lasehinde, M. K. Jayanthi, Ramith Ramu

**Affiliations:** ^1^Department of Biotechnology and Bioinformatics, JSS Academy of Higher Education and Research, Mysuru, Karnataka, India; ^2^Department of Pharmacology, JSS Medical College, JSS Academy of Higher Education and Research, Mysuru, Karnataka, India; ^3^Department of Restorative Dental Science, College of Dentistry, King Saud University, Riyadh, Saudi Arabia; ^4^Department of Biology, Washington University, St. Louis, MO, United States

**Keywords:** probiotics, lactic acid bacteria, α-glucosidase, α-amylase, *in silico*

## Abstract

Probiotics are live microorganisms with various health benefits when consumed in appropriate amounts. Fermented foods are a rich source of these beneficial organisms. This study aimed to investigate the probiotic potential of lactic acid bacteria (LAB) isolated from fermented papaya (*Carica papaya* L.) through *in vitro* methods. The LAB strains were thoroughly characterized, considering their morphological, physiological, fermentative, biochemical, and molecular properties. The LAB strain's adherence and resistance to gastrointestinal conditions, as well as its antibacterial and antioxidant capabilities, were examined. Moreover, the strains were tested for susceptibility against specific antibiotics, and safety evaluations encompassed the hemolytic assay and DNase activity. The supernatant of the LAB isolate underwent organic acid profiling (LCMS). The primary objective of this study was to assess the inhibitory activity of α-amylase and α-glucosidase enzymes, both *in vitro* and *in silico*. Gram-positive strains that were catalase-negative and carbohydrate fermenting were selected for further analysis. The LAB isolate exhibited resistance to acid bile (0.3% and 1%), phenol (0.1% and 0.4%), and simulated gastrointestinal juice (pH 3–8). It demonstrated potent antibacterial and antioxidant abilities and resistance to kanamycin, vancomycin, and methicillin. The LAB strain showed autoaggregation (83%) and adhesion to chicken crop epithelial cells, buccal epithelial cells, and HT-29 cells. Safety assessments indicated no evidence of hemolysis or DNA degradation, confirming the safety of the LAB isolates. The isolate's identity was confirmed using the 16S rRNA sequence. The LAB strain *Levilactobacillus brevis* RAMULAB52, derived from fermented papaya, exhibited promising probiotic properties. Moreover, the isolate demonstrated significant inhibition of α-amylase (86.97%) and α-glucosidase (75.87%) enzymes. *In silico* studies uncovered that hydroxycitric acid, one of the organic acids derived from the isolate, interacted with crucial amino acid residues of the target enzymes. Specifically, hydroxycitric acid formed hydrogen bonds with key amino acid residues, such as GLU233 and ASP197 in α-amylase, and ASN241, ARG312, GLU304, SER308, HIS279, PRO309, and PHE311 in α-glucosidase. In conclusion, *Levilactobacillus brevis* RAMULAB52, isolated from fermented papaya, possesses promising probiotic properties and exhibits potential as an effective remedy for diabetes. Its resistance to gastrointestinal conditions, antibacterial and antioxidant abilities, adhesion to different cell types, and significant inhibition of target enzymes make it a valuable candidate for further research and potential application in the field of probiotics and diabetes management.

## Introduction

Diabetes mellitus is one of the multiple widespread metabolic disorders. The disorder is characterized by increased blood glucose levels (hyperglycemia), which is a result of insufficient production or improper function of insulin (Ramu et al., 2022; Sreepathi et al., 2022). The deregulation of the carbohydrate hydrolyzing enzymes (α-amylase and α-glucosidase) and insulin hormone effectively leads to chronic diabetes mellitus (Telagari and Hullatti, 2015; Ramu and Patil, 2021; Patil et al., 2022a). Studies also prove that altered gut microbiota effectively influences glucose homeostasis (Andersson et al., 2010; Zhang et al., 2014, 2016; Yan et al., 2019; Cunningham et al., 2021). Among many therapeutic approaches like gluconeogenesis inhibition, insulin injection, and increasing the number of glucose transporters to manage hyperglycemia (Forouhi et al., 2018), reduction in gastrointestinal glucose production and absorption through inhibition of carbohydrate hydrolyzing enzymes could be considered as a potent recourse. Inhibition of α-amylase and α-glucosidase present in the brush border of the small intestine can significantly decrease the increased blood glucose level by delaying glucose absorption after a mixed diet (Adelusi et al., 2014). There are synthetic drugs identified for their inhibitory profiles against carbohydrate hydrolyzing enzymes that are associated with the after effects for its long-term consumption (Santos and Silva, 2020). As a way out from synthetic drugs, functional foods were introduced that possess the ability to provide energy along with remarkable medicinal properties. Food replacing medication that promotes health status and prevents risk factors that are responsible for diseases is a new approach to nutrition science to treat chronic diseases (Ramu et al., 2016a,b). Functional foods also take part in addressing the life expectancy and desire of people toward improved quality of life (Nguyen et al., 2019). Functional foods are not merely a meal or nutrient supplements that only provide basic nutrition but also a source for physical and mental wellbeing. In this regard, probiotics (live microbes) are functional foods when administered in an adequate amount confer health benefits (Syngai et al., 2015). Most of the probiotics available are from the fermented source, such as vegetables, fruits, and dairy products. Scientific studies support the effectiveness of probiotics from dairy sources as potent antidiabetic agents (Sujaya et al., 2016; Widodo et al., 2019; Chaudhary and Mudgal, 2020; Kinariwala et al., 2020) by modulating the altered gut microbiota. Yet, dairy products are associated with limitations such as cholesterol and lactose intolerance (Priyanka et al., 2019; Huda et al., 2021). Thus, the isolation of *Lactobacillus* spp. from fermented fruits with effective antioxidant and inhibitory abilities would be a reliable approach to address diabetes. This property is of great significance in case of diabetes as it inhibits the development of tissue damage caused due to oxidative stress, resulting in the progression of diabetes mellitus (Negahdari et al., 2021). In this regard, any probiotic species used in the treatment of diabetes mellitus should possess inhibitory activity against the carbohydrate hydrolyzing enzyme and free radical-associated damage. In addition to the obvious antidiabetic property, because probiotics are ingested as a meal or a part of the meal, the isolated LAB strains need to possess instinctive tolerance to phenol, acid bile and gastrointestinal juice conditions, restoration of mucosal integrity, fabrication of enzymes and vitamins, antibiotic sensitivity, and antagonistic activities.

Papaya (*Carica papaya* L) of Caricaceae family is a fruit with a low glycemic index, rich in fiber content, with additional properties such as an ability to improve intestinal motility, and an excellent adjuvant in combined therapies against Alzheimer's and allergic reactions. The consumption of fermented papaya is found to exhibit excellent antioxidant (Leitão et al., 2022), anticancer (Logozzi et al., 2020), and antidiabetic properties (Raffaelli et al., 2015). Since all the beneficiary nutrients of the fruit are highly supported with its pleasing taste, the fruit can be considered as a unique food matrix to carry probiotics to all age groups with a convincing taste (Perricone et al., 2015). Based on these findings, the objective of this study was to isolate LAB from non-dairy sources and evaluate their *in vitro* and *in silico* properties, and their potential antidiabetic activity by inhibiting carbohydrate hydrolyzing enzymes (α-amylase and α-glucosidase).

## Materials and methods

### Materials and instruments

The chemicals that are used in the present study were purchased from Himedia Laboratories Pvt. Ltd., Mumbai, India. The pathogens *Bacillus subtilis* (MTCC 10403), *Escherichia coli* (MTCC 443), *Pseudomonas aeruginosa* (MTCC 424), *Micrococcus luteus* (MTCC 1809), *Staphylococcus aureus* (MTCC 1144), *Salmonella enterica typhimurium* (MTCC 98), *Klebsiella pneumonia* (MTCC 10309), *Bacillus cereus* (MTCC 1272), *Klebsiella aerogenes* (MTCC 2822), and *Pseudomonas fluorescens* (MTCC 667) were received from Microbial Type Culture Collection (MTCC) and Gene Bank, Chandigarh, India. In the current study, the instruments used were microplate reader (Multiskan FC, Thermo Fisher Scientific, Mumbai, India), centrifuge (Remi CPR-30 plus, Mumbai, India), Vortex Shaker (SPINIX, Kolkata, India), PVDF syringe filter (0.22 μm; Thermo Fisher Scientific, Mumbai, India), CO_2_ incubator (Thermo Fisher Scientific, Mumbai, India), and shaking incubator (Thermo Fisher Scientific, Mumbai, India).

### Sample preparation, isolation, and cell preparation

Fresh raw papaya was purchased from the local seller in Mysuru, Karnataka. The papaya pieces were washed with lukewarm water (32.5°C) and grated, and table salt (1 tablespoon per kilo of papaya) was added, covered, and allowed to ferment in shaking incubator 120 rpm, 48 h, pH 6.5 at 37°C. Furthermore, 1 g of this mixture was serially diluted to isolate LAB on MRS agar plates at 37°C for 24 h (Swain et al., 2014). The individual colonies that were formed on MRS agar were isolated and subcultured by the streak plate method and purified with three successive subcultures. The isolated colonies were considered primary cultures, and glycerol stock (15% glycerol at −20°C) was prepared for the same to carry out future studies. For further assays, the cell suspension (1 × 10^8^ CFU/mL), intact cells (IC), cell-free extract (CFE) obtained by breaking down bacterial cells to extract their internal components, and cell-free supernatant (CFS) are obtained by separating bacterial cells from the liquid part of a bacterial culture which were prepared as mentioned in the previous study (Kumari et al., 2022).

### Biochemical assay

The pure isolates were subjected to preliminary assessments of LAB by cell morphology, gram staining, and catalase test as mentioned by Reuben et al. (2019). To assess the temperature and pH tolerance, the isolate was subjected to varying temperatures (4, 15, 37, 40, and 50°C) and pH 2 (extreme acidic condition), pH 4 (normal pH range of the skin and mucous membranes), pH 6 (slightly acidic body fluids), and pH 7.4 (physiological pH of blood). Salt tolerance was also assessed at 2, 3, 6, and 10% NaCl concentration in MRS broth. Furthermore, to check the ability of the isolates to utilize carbohydrates, carbohydrate fermentation was performed using different sugars (arabinose, fructose, sorbitol, lactose, maltose, xylose, sucrose, mannitol, galactose, glucose, and lactose) (Divyashree et al., 2021). The other biochemical tests such as citrate utilization, methyl red, Voges-Proskauer, indole, starch hydrolysis, and gelatin liquefaction tests were also performed as per the protocol mentioned by Kefir et al. (2018).

### Probiotic properties

#### Acid bile salt tolerance

The isolate was subjected to acid and salt tolerance assay as per the protocol of Kusada et al. (2021) with minor modifications. The method briefly involved 24 h culture to be tested in MRS broth (pH 2.0) with 0.3% and 1% ox gall supplementation separately to determine the tolerance of the isolates. After 0, 2, and 4 h of inoculation, the cell suspensions from each concentration of ox gall were plated on MRS agar and incubated (24 h, 37°C) to get the count of the cells that survived. The formula given below is used to calculate the survival rate.


$$\begin{array}{l} \text{Survival rate }\left(\text{\%}\right)=\frac{\text{Biomass at time }\left(\text{t}\right)}{\text{Biomass at initial time }\left(0\right)}\;\times \;100 \end{array}$$

#### Phenol tolerance assay

On conducting phenol tolerance assay, the isolate was inoculated into MRS broth provided with varying concentrations of phenol (0.1, 0.4%) and incubated for 24 h, and plating was performed for above suspensions on MRS agar at 0 h and 24 h. Plates were incubated at 37°C for 24 h to evaluate the survival rate (Goh et al., 2021).


$$\begin{array}{l} \text{Survival rate }=\text{ log}10\;\left(\text{initial population}\right)\;-\text{ log}10\;\left(\text{finalpopulation}\right) \end{array}$$

#### Cell surface hydrophobicity

Cell surface hydrophobicity was performed to know the ability of the isolate to adhere to the organic solvents as described by Ohn et al. (2020) with slight modifications. This is an indirect approach to know the ability of the isolate to adhere to the intestinal cells. In brief, the overnight culture was subjected to centrifugation (500 × g, 3 min, 4°C), leading to the separation of cells. After washing with PBS twice, the cells were re-suspended in the same buffer. The OD value of the above suspension was adjusted to 1.0 absorbance in three tubes, and an equal volume of non-polar (xylene, chloroform, and ethyl acetate) solvents was added and vortexed 2 min before incubating at room temperature (28°C) for 30 min. After layer separation, the absorbance of non-polar solvent and suspension was read at 600 nm.


$$\begin{array}{l} \text{Hydrophobicity }\left(\text{\%}\right)=\frac{\left[{\text{A}}_{\text{suspension}}\;-\;{\text{A}}_{\text{solvent}}\right]}{{\text{A}}_{\text{solvent}}} \end{array}$$

where A_suspension_ is the absorbance of LAB suspension after layer separation, and A_solvent_ is the absorbance of organic solvent after layer separation.

### Autoaggregation

The ability of the isolates to autoaggregate was assessed as per the protocol described by Divyashree et al. (2021) with minimum modifications. In brief, the overnight incubated cultures were centrifuged at 3,500 × g for 10 min at 4°C. The obtained pellets were washed with PBS twice and re-suspended in the same, followed by vortexing for 10 s. The suspension was then incubated at 37°C, and the top most layer was evaluated at 0, 2, 4, 6, 10, and 24 h using a microplate reader at an absorbance of 600 nm. Autoaggregation was calculated using the equation below.


$$\begin{array}{l} \text{Autoaggregation }\left(\text{\%}\right)=\frac{\left[{\text{A}}_{0}-{\text{A}}_{\text{t}}\right]}{{\text{A}}_{0}}\;\times \;100 \end{array}$$

where A_0_ is the absorbance at time “0 h”, and A_t_ is the absorbance at time “t” (2, 4, 6, 10, and 24 h).

### Coaggregation

The sample preparation for coaggregation is similar to that of autoaggregation assay. It is followed by mixing the isolate with five different pathogens (*Escherichia coli* MTCC 4430, *Micrococcus luteus* MTCC 1809, *Bacillus subtilis* MTCC 10403, *Salmonella enterica typhimurium* MTCC 98, and *Pseudomonas aeruginosa* MTCC 424) and incubated for 120 min at 37°C. Furthermore, the absorbance was measured at 600 nm using microplate reader, and percentage of coaggregation was calculated using the equation below.


$$\begin{array}{l} \frac{\left({\text{A}}_{\text{LAB}}\;+\;{\text{A}}_{\text{Pathogen}}\right)-{\text{A}}_{\text{mix}}}{{\text{A}}_{\text{LAB}}+{\text{A}}_{\text{Pathogen}}}\;\times \;100 \end{array}$$

where A_LAB_ + A_Pathogen_ is the absorbance of LAB and pathogen mixture at 0 h, and A_mix_ is the absorbance after 2 h of incubation.

### Gastric juice tolerance assay

To mimic intestinal and gastric juice, trypsin (1 g/L) and pepsin (3 g/L) were dissolved in phosphate buffer saline at pH 8 and pH 3, respectively, and filter of size 0.22 μm was used for sterilization. The isolates (10^8^ CFU/mL) were suspended in sterilized gastric juice and incubated at 37°C for 0, 1, 3, and 6 h in 5% CO_2_ incubator. After incubation for 3 h with gastric juice, the isolates were added to the stimulated intestinal juice and incubated for 1, 3, 5, and 7 h. Furthermore, the gastrointestinal tolerant isolate was assessed by counting viable colonies using the colony counter after plating all the above suspension by spread plate technique on MRS agar. The equation below is used in computing the survival rate (Repally et al., 2017).


$$\begin{array}{l} \text{Survival rate }\left(\text{\%}\right)=\frac{\left(\text{Log CFU }{\text{N}}_{\text{1}}\right)}{\left(\text{Log CFU }{\text{N}}_{0}\right)}\times \;100 \end{array}$$

where N_1_ is the total number of cells survived at different incubation time, and N_0_ is the total number of living cells before treatment.

### Antibacterial activity

Disk diffusion method was used to check the antibacterial activity of the isolate RAMULABB52 against 10 different pathogens, namely *Bacillus subtilis* (MTCC 10403), *Escherichia coli* (MTCC 443), *Pseudomonas aeruginosa* (MTCC 424), *Micrococcus luteus* (MTCC 1809), *Staphylococcus aureus* (MTCC 1144), *Salmonella enterica typhimurium* (MTCC 98), *Klebsiella pneumonia* (MTCC 10309), *Bacillus cereus* (MTCC 1272), *Klebsiella aerogenes* (MTCC 2822), and *Pseudomonas fluorescens* (MTCC 667) as per the protocol of Balouiri et al. (2016).

### Antibiotic sensitivity

The antibiotic sensitivity of the LAB isolate was evaluated by disk diffusion method as described by El Issaoui et al. (2021). In brief, the isolated LAB strain was inoculated on MRS agar media by spread plate method, followed by placing the disk loaded with antibiotics chloramphenicol (30 mcg/disk), clindamycin (2 mcg/disk), kanamycin (30 mcg/disk), vancomycin (30 mcg/disk), streptomycin (100 mcg/disk), methicillin (5 mcg/disk), cefixime (5 mcg/disk), gentamicin (10 mcg/disk), ampicillin (10 mcg/disk), tetracycline (30 mcg/disk), erythromycin (15 mcg/disk), rifampicin (5 mcg/disk), and azithromycin (15 mcg/disk) and incubated (37°C, 24 h). Using CLSI (2018) (Chang, 2018) scale, the zone of inhibition was measured and interpreted as resistant and susceptible.

### Molecular characterization of LAB

The DNA of the isolate was extracted, and molecular characterization was performed using 27F [5′AGA GTTTGATCCTGGCTCAG3′] and 1492R [5′GGTTACCT TGTTACGACTT3′] primers to amplify 16s rRNA (ribosomal RNA) sequence, amplified, and sequenced. The homology search of the isolate was performed using Basic Local Alignment Search Tool (BLAST), and the obtained sequence was deposited in the GenBank sequence database to get the accession number (Borovic et al., 2015).

### *In vitro* adhesion to chicken crop epithelial cells

To know the ability of the isolate to adhere with chicken crop epithelial cell under *in vitro* condition, the present assay was performed as per the protocol described by Somashekaraiah et al. (2019). In brief, 10^8^ CFU/mL isolates were incubated for 30 min at 37°C with chicken crop epithelial cells (1 × 10^6^ cells/mL). The non-adherent cells were eliminated by centrifugation (500 × g for 3 min, 4°C). The obtained pellets were re-suspended in 100 μL of PBS (sterile) and stained with crystal violet for microscopic observation (Olympus BX3M, Evident Scientific Private Limited, Haryana, India).

### *In vitro* adhesion to buccal epithelial cells

The methodology adopted to check the ability of the isolates to adhere with the buccal epithelial cells was similar to the previous assay with slight modification. In brief, the collected buccal epithelial cells were washed using saline twice and centrifuged at 1,300 × g for 3 min at 4°C. The pellets obtained were rinsed thrice using sterile PBS and suspended in to sterile saline. Now, 100 μL of LAB isolates (10^8^ CFU/mL) were incubated for 30 min with 400 μL (3 × 10^6^ CFU/mL) of buccal epithelial (diluted) cells at 37°C. Furthermore, the cells were stained with crystal violet and assessed for their ability to adhere to buccal epithelial cells (Dicks et al., 2014).

### *In vitro* adhesion to HT-29 cells, culture, and growth conditions

The adhesion ability of the isolate with the human colon adenocarcinoma cell line (HT-29) was evaluated as per the methodology followed by Fonseca et al. (2020). The HT-29 cells were collected from National Center for Cell Sciences (NCCS, Pune, India), the cells were cultured in sodium pyruvate-free Dulbecco's Modified Eagle Medium (DMEM) with GlutaMax (Gibco, UK) at 37°C in carbon dioxide (5%) incubator. Furthermore, 10% (v/v) FBS, 100μg/mL of streptomycin, and penicillin were added into the media. Now, the HT-29 cells were subcultured into six-well culture plates containing sterile media and incubated at 37°C with a CO_2_ atmosphere until the inoculated cells divided and confluxes 80% of the prOvided media. To enhance cell proliferation and constantly maintain the nutrient supply, the culture media was changed in an alternate day cycle.

The isolate was subcultured in MRS broth at 37°C for 24 h and used in the assay. The HT-29 cells of concentration 10^8^ CFU/mL were put back into DMEM and washed using PBS. 1 mL of bacterial suspension was mixed with HT-29 cells in the well and incubated for 30 min and 1h (37°C, 5% CO_2_). After the incubation period, about 1mL of 0.1% Triton-X solution (in phosphate buffer saline) was added to the cell mixture to lyse the cells, followed by removal of non-adherent LAB. Furthermore, adhered cells were rinsed using 1,000 μL of PBS. After 10 min, the solution with detached bacteria was serially diluted and plated on sterile MRS agar. These plates were incubated at 37°C for 24 h. The adhesion ability of our isolates was measured by comparing the ratio of cells seeded initially with that of the cells obtained after the washing step. To get the stable result, the present experiment was performed in duplicates thrice (Srikham and Thirabunyanon, 2022).

### Hemolytic assay

Hemolytic activity of the isolate was carried out as per the protocol of Yasmin et al. (2020). The isolate used in the assay was subcultured on blood agar media containing 5% sheep blood. The lysis of red blood cell (RBC) in the media surrounding the colonies was observed for hemolytic activity. The zone formation is classified into three forms: γ-hemolysis (which is considered safe, as there will be no formation of zones around colonies), α-hemolysis (formation of green zones around colonies), and β-hemolysis (clear zone around colonies).

### DNase activity

The DNase activity of the LAB strain was evaluated by inoculating the isolate on DNase agar plate by streak plate method and incubated at 37°C for 48 h. After incubation, the plates were observed for the formation of zones around the colonies. The formation of prominent red colored zone around the colonies indicates DNase activity (Santos and Silva, 2020).

### Antioxidant activity

2,2-Diphenyl-1-Picrylhydrazyl (DPPH) scavenging activity of the isolate was assessed as per the protocol described by Nakagawa and Miyazaki (2017) with modifications. In brief, 50 μL of the prepared DPPH (40 mg DPPH in 1000 mL of methanol) was added to a 25 μL cell suspension (10^3^, 10^6^, and 10^9^ CFU/mL), and the volume is made up to 200 μL using methanol. The reaction mixture was incubated at 37°C for 30 min in a dark room. Absorbance was measured at 517 nm using a spectrophotometer. The 2,2′-azino-bis (3-ethylbenzothiazoline-6-sulfonic acid) (ABTS) scavenging activity of the isolate is performed as per the protocol described by Lin et al. (2018) with modification. In brief, 120 μL of ABTS is mixed with 80 μL of cell suspension (10^3^, 10^6^, and 10^9^ CFU/mL). The reaction mixture is incubated at room temperature (28°C) for 30 min. Absorbance was read at 734 nm using a spectrophotometer. Radical scavenging activity of the isolate with DPPH and ABTS is computed using the formula below.


$$\begin{array}{l} \text{Scavenging activity }\left(\text{\%}\right)=\frac{\left[1-{\text{A}}_{\text{s}}\right]}{{\text{A}}_{\text{c}}}\times \;100 \end{array}$$

where A_s_ is the absorbance of sample, and A_c_ is the absorbance of control.

### Inhibitory activity of the enzymes α-glucosidase and α-amylase

The inhibitory activity of α-glucosidase was carried out as per Kwun et al. (2020) with slight modification. In this study, to determine the ability of the isolate to inhibit α-glucosidase, the prepared 100 μL of IC, CFE, and CFS were mixed separately with 700 μL of potassium phosphate buffer (pH 6.8, 50 mM) and incubated for 10 min at 28°C. Furthermore, 100 μL of α-glucosidase (0.25 U/mL) was added to the mixture prepared in the previous step, and incubation was continued for the next 15 min at 37°C. Now, 5 mM p-nitrophenyl-D-glucopyranoside (pNPG) of 100 μL was added to the previous step mixture and incubated at the same temperature for a span of 30 min. To stop the enzyme activity, approximately 1 mL of Na_2_CO_3_ (0.1M) was added. Using a microplate reader, the OD was measured at 405 nm (Ramu et al., 2014).

In addition, the inhibitory activity of the isolate against α-amylase was carried out using porcine pancreatic amylase. In brief, about 500 μL of IC, CFE, and CFS were mixed with 500 μL of 0.1 M phosphate buffer saline PBS (pH 7.2) having α-amylase of concentration 0.5 mg/mL and incubated for 10 min at 25°C. After incubation, 500 μL of 1% starch (starch in PBS 0.1 M, pH 7.2) mixture was added to each reaction tube and incubated for 10 min at 25°C. The reaction was stopped by adding 1 μL of 3, 5-dinitrosalicylic acid (DNS). Furthermore, the reaction mixture was placed in a water bath at 60°C for 5 min. The reaction mixtures were brought back to room temperature (28°C) and diluted (distilled water of 10 mL). The absorbance of diluted samples was observed at 540 nm (Ramu et al., 2015).

The formula given below is used to calculate the inhibitory activity of isolates.


$$\begin{array}{l} \text{Inhibition }\left(\text{\%}\right)=\frac{\left({\text{A}}_{\text{c}}\;-\;{\text{A}}_{\text{S}}\right)}{{\text{A}}_{\text{c}}}\times \;100 \end{array}$$

where A_s_ is the absorbance of (enzyme + sample), and A_c_ is the absorbance of enzyme without sample.

### Extraction of organic acids

To extract organic acid from selected LAB, the protocol described by Nuryana et al. (2019) was adopted with slight modification. In brief, the LAB strain (0.5 mL) was inoculated into screw cap test tube containing MRS broth (5 mL) and incubated for 5 days at 37°C with 150 rpm agitation in shaking incubator. Furthermore, the sampling process was continued by centrifuging the tubes at 5,500 × g for 20 min at 4°C. After obtaining the supernatant, it was further analyzed using LC-MS with the following conditions: 0.5 mL of the filtered supernatant was injected into an LC-MS/MS analyzer for organic acid analysis. The mobile phase consisted of 0.01 mM ammonium acetate: acetonitrile (NH_4_OAc: CH_3_CN; 50:50, pH 8) and CH_3_CN with 0.05% formic acid (HCOOH) to evaluate the extracted organic acids (Kumari et al., 2023).

### *In silico* studies

#### Pass pharmacological analysis

The pharmacological activity of the organic acid compound was evaluated by PASS (online server), which draws out the specific pharmacological activity of the input compound (Ganavi et al., 2022). The collected data were computed and classified as probability activity (Pa) and probable inactivity (Pi). The compound with greater Pa values than Pi is considered operable for specific pharmacological activity (Pogodin et al., 2018; Gurupadaswamy et al., 2022).

#### Molecular docking simulation

The 2D structure of the organic acids derived from supernatant was drawn using ChemSketch, and 3D optimization was performed using ACD ChemSketch. Furthermore, for the purpose of docking, the ligand was prepared using AutoDock 4.2 (Jyothi et al., 2022; Martiz et al., 2022a,b). Acarbose was used as a positive control (Kumar et al., 2021).

The protein sequence of α-glucosidase (*Saccharomyces cerevisiae*) MAL-32 was obtained from UniProt ID: P38158 for the construction of homology model (Patil et al., 2022a,b). The template search was performed before the construction of protein model to identify the highest percentage of alignment among Protein Data Bank (PDB) which is very close to the homology model. Furthermore, the homology model of the target protein was built using SWISS-MODEL (https://swissmodel.expasy.org/) which is based on the homology structure α-glucosidase of *S. cerevisiae* (PDB ID: 3AXH) with identical (72%) and similar (84%) sequence at 1.8 Å resolution (Waterhouse et al., 2018), and the protein model constructed and used in this study is identical with previous investigation (Patil et al., 2022c,d). The x-ray crystal structure of the second target (α-amylase, PDB ID: 1DHK) was retrieved using RCSB PDB database (https://www.rcsb.org/).

The preparation of protein, ligand, and virtual screening of compounds was performed using AutoDock tools 1.5.6 based on previous studies (Patil et al., 2021a,b; Sajal et al., 2022). Furthermore, binding site of α-amylase and α-glucosidase was predicted by a literature survey (Bompard-Gilles et al., 1996; Patil et al., 2021a). The grid box of size 40 Å × 40 Å × 40 Å containing binding pocket in the position x = −17.489 Å, y = −8.621 Å, and z = −19.658 Å was constructed for α-glucosidase. Concurrently, a grid box of size 40 Å × 40 Å × 40 Å containing the binding pocket in the position x = 103.469 Å, y = 37.176 Å, and z = 19.607 Å was constructed for α-amylase (Patil et al., 2021b). Using AutoDock Vina software, the interaction between target proteins (α-glucosidase and α-amylase) and ligands (organic acids derived from LAB) has been studied (Kumar et al., 2022; Patil et al., 2022d,e).

#### Molecular dynamic simulation

After docking, the obtained lead compound with high binding affinity, non-bonded interaction, and hydrogen bond was subjected to MD simulation, to get a clear instinct of structures at an atomic level, its behavior, stability, and conformational changes that take place during complex formation using the trajectories in terms of RMSD, RMSF, Rg, SASA, and the number of ligand hydrogen bonds (Martiz et al., 2022b,c; Patil et al., 2022f).

The stability of the complex against a given time is evaluated using RMSD. To understand the binding efficiency of hydroxy citric acid with the target protein, RMSF analysis was conducted. Furthermore, to know the possible change in the structure of the protein, during complex formation, Rg plot was analyzed (Maradesha et al., 2023; Patil et al., 2023). The SASA plot was evaluated to predict the conformational change in the binding region (Pradeep et al., 2022). Furthermore, the ligand hydrogen bonds were analyzed to know the structural rearrangements, based on the plot obtained it can be said that the complex may have undergone structural modification (Maradesha et al., 2022a,b).

The simulation for the chosen compound was performed for 100 ns time scale using bimolecular software GROMACS-2018.1 (Pushpa et al., 2022). To obtain the protein parameters, CHARMM36 force field was applied and Swiss PARAM server was used to obtain ligand topology (Zoete et al., 2011). To maintain the neutrality and salt concentration (0.15 M) of the entire system, Na+ and Cl- counter ions were added and continued with energy minimization using steepest descent method of 50,000 steps (Martiz et al., 2022a,b). Furthermore, equilibration of the system was performed by using NTP and NVT ensemble classes at 310K temperature and 1 bar pressure. Furthermore, using XMGRACE software, the graph was plotted for all the above trajectories (Martiz et al., 2022c).

#### Binding free energy calculations

The binding free energy of the formed complex was calculated by considering the tree components, i.e., molecular mechanical energy, polar and a polar solvation energies, and estimated using Molecular Mechanics/Poisson–Boltzmann Surface Area (MM-PBSA) approach using g_mmpbsa program (GROMACS plugin) (Patil et al., 2021a,b; Shivanna et al., 2022). For the calculation of binding free energy of the complex (protein–ligand), the last 50 ns frames which were extracted from MD trajectory were utilized (Martiz et al., 2022c; Nivetha et al., 2022).

### Statistical analysis

All the experiments were carried out in triplicates, and the standard deviation is displayed in error bars on graph (GraphPad Prism Software Inc., San Diego, CA, USA). Data were examined using ANOVA, and differences were considered substantial at *p-value of* ≤ 0.05 (Ramu et al., 2014, 2015).

## Results

### Preliminary characterization of LAB strain

In this study, the selection criteria involved identifying seven distinct colonies, all of which were either gram-negative and catalase positive, except for one colony that exhibited gram-positive and catalase-negative characteristics. The isolate selected was rod-shaped, gram-positive, and catalase-negative, with an ability to partially grow at 15 and 40°C with an optimal growth at 37°C. The isolate demonstrated a tolerance up to 3% NaCl and different pHs (2, 4, 6, and 7.4), with an optimal growth observed at pH 7.4. The isolate could ferment carbohydrates (arabinose, fructose, sorbitol, maltose, sucrose, mannitol, galactose, glucose, and lactose). As a result of fermentation, the formation of acid was observed in the reaction tubes. Furthermore, the isolate was positive to methyl red test and negative for indole, Voges-Proskauer, citrate utilization, starch hydrolysis, and gelatin liquefaction tests (Table 1).

**Table 1:** Phenotypic characterization, fermentation ability, and biochemical properties of LAB strain isolated from fermented papaya.

**Test**		**Isolate**
		**RAMULAB52**
Preliminary tests	Gram staining	+
	Morphology	Rod
	Catalase	–
Temperature tolerance		
	4°C	–
	15°C	∂
	37°C	+
	40°C	∂
	50°C	–
Salt tolerance		
	2%	+
	3%	+
	6%	–
	10%	–
pH tolerance		
	2	+
	4	+
	6	+
	7.4	+
Carbohydrate fermentation		
Monosaccharide sugars	Arabinose	+
	Fructose	+
	Sorbitol	+
	Mannitol	+
	Galactose	+
	Xylose	–
	Glucose	+
Disaccharide sugars	Lactose	+
	Maltose	+
	Sucrose	+
	Lactose	+
IMViC test		
	Methyl red test	+
	Indole test	–
	Citrate utilization test	–
	Voges-Proskauer test	–
	Gelatin liquefaction test	–
	Starch hydrolysis test	–

“+” indicates growth, “–” indicates absence of growth, and ‘∂' indicates partial growth.

### Acid bile salt tolerance

**Figure 1:**
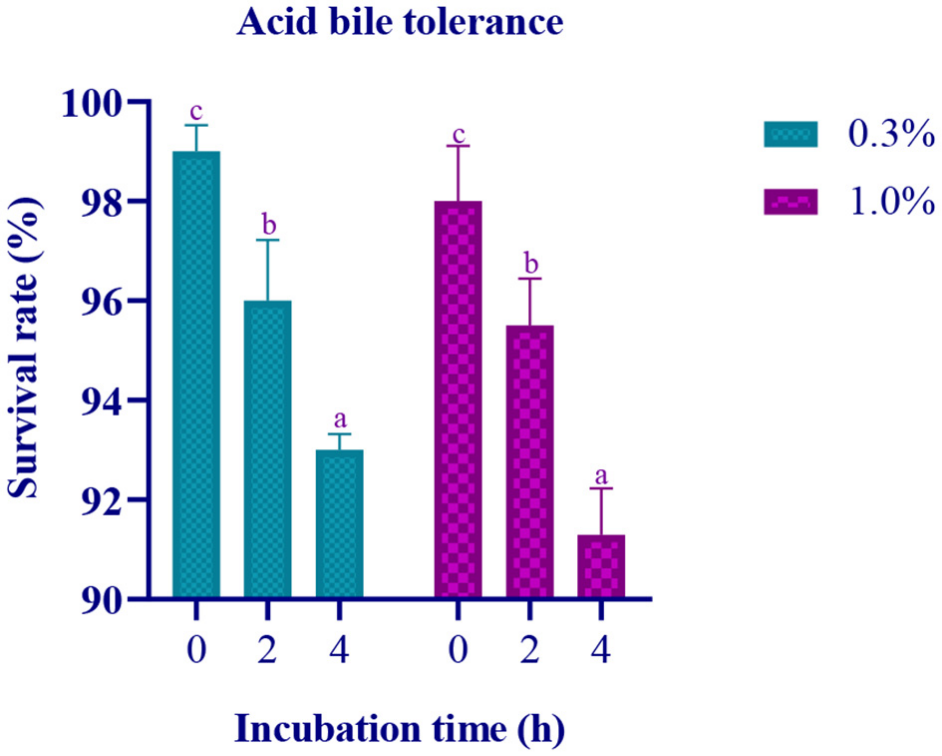
Data on survival rate of the LAB isolate at pH 2 with varying concentration of Ox gall (0.3% and 1%) against incubation time (0, 2, and 4 h) is expressed in mean ± standard deviation with significantly different *p* ≤ 0.05 as per Duncan multiple range test and mentioned with (a, b, and c) superscripts.

In this study, the isolate's survival rate was evaluated under different conditions, including varying concentrations of acid bile (0.3% and 1%), incubation times (0, 2, and 4 h), and after incubation at 37°C for 24 h. The results showed that the isolate exhibited a survival rate ranging from 93% to 99% at 0.3% acid bile concentration and from 91% to 98% at 1% acid bile concentration in MRS media (Figure 1).

### Phenol tolerance assay

The study found that the isolate had the ability to survive and grow in the presence of phenol up to a concentration of 0.4%. The growth rate was similar to that of the control group without phenol. However, the survival rate of the isolate was observed to decrease by 0.25% at the concentration of 0.4% phenol when compared to blank (Table 2).

**Table 2:** Survival rate of the isolate at different concentrations of phenol and cell surface hydrophobicity of LAB isolate.

**Phenol tolerance Log (CFU/mL)**						**Hydrophobicity (%)**		
**Blank (without phenol)**		**0.1 %**		**0.4 %**		**Xylene**	**Chloroform**	**Ethyl acetate**
**0 h**	**24 h**	**0 h**	**24 h**	**0 h**	**24 h**	**30 min**	**30 min**	**30 min**
8.45 ± 0.12	8.56 ± 0.23	8.36 ± 0.01	8.15 ± 0.02	7.35 ± 0.05	7.10 ± 0.02	72.25 ± 0.89	66.09 ± 0.54	0.0 ± 0.0

### Determination of cell surface hydrophobicity

The cell surface hydrophobicity of the isolate was evaluated using non-polar solvents, namely xylene, chloroform (acidic), and ethyl acetate (basic), and the respective values were obtained as 72.25 ± 0.89 and 66.09 ± 0.54 for xylene and chloroform, indicating significant hydrophobicity due to the presence of hydrophobic components in the outer layer of the cell wall. However, no hydrophobicity was observed for the isolate with ethyl acetate, and the value obtained was 0.0 ± 0.0 (Table 2).

### Bacterial autoaggregation and coaggregation abilities

**Figure 2:**
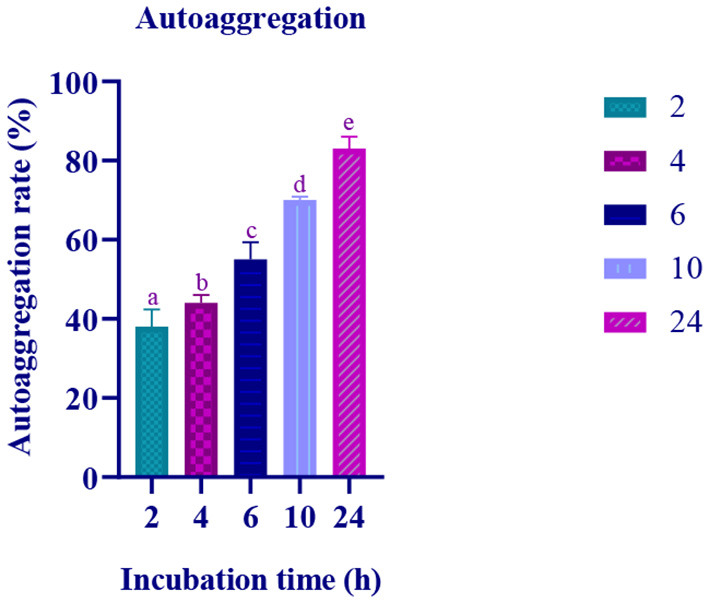
Autoaggregation ability of the LAB isolate at varying time intervals (2, 4, 6, 10, and 24 h) is expressed in mean ± standard deviation with significantly different *p* ≤ 0.05 as per Duncan multiple range test and mentioned with (a, b, c, d, and e) superscripts.

**Figure 3:**
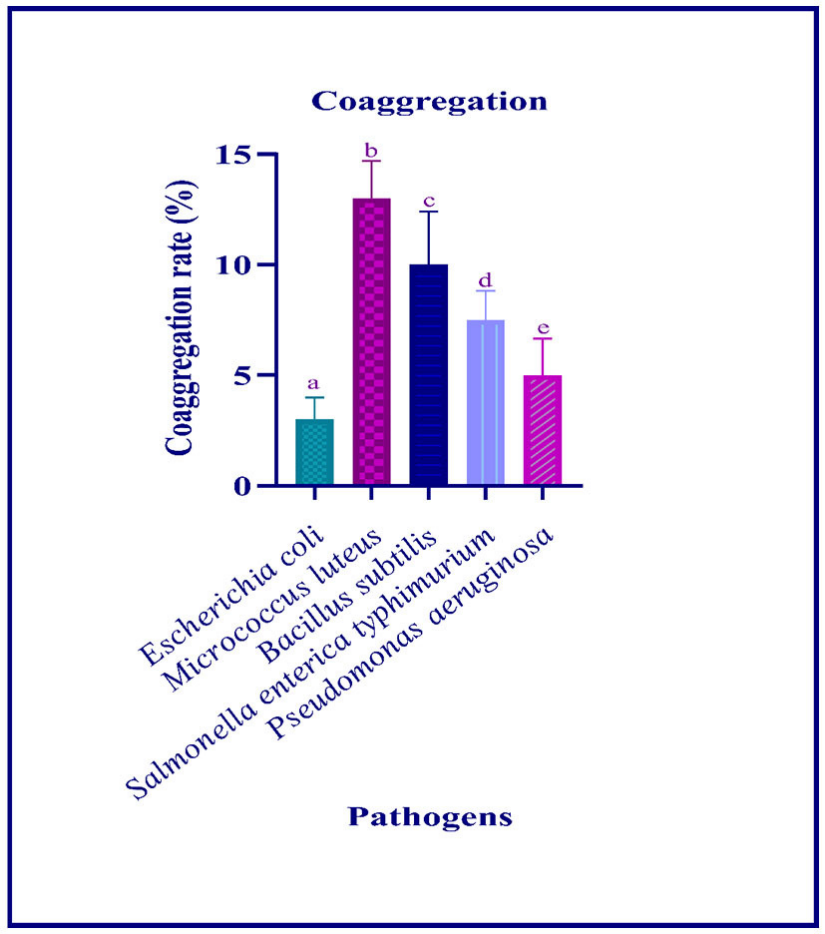
Coaggregation ability of the LAB isolate with different pathogens is expressed in mean ± standard deviation with significantly different *p* ≤ 0.05 as per Duncan multiple range test and mentioned with (a, b, c, d, and e) superscripts.

The autoaggregation of the isolate increased with progression in the incubation period. At the last hour (24 h) of incubation, the autoaggregation of the isolate was found to be 83.06% (Figure 2). The coaggregation of the isolate with pathogen *Micrococcus luteus* (MTCC 1809) was high, and moderate coaggregation is observed with *Escherichia coli* (MTCC 443), Bacillus subtilis (MTCC 10403), *Salmonella enteric typhimurium* (MTCC 98), and *Pseudomonas aeruginosa* (MTCC 667) pathogens (Figure 3).

### Gastric juice tolerance assay

**Figure 4:**
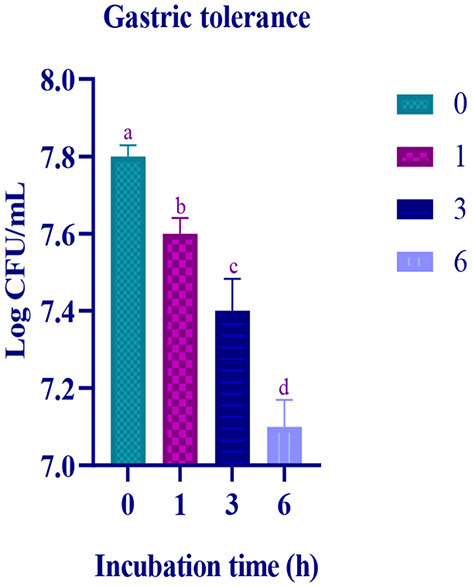
Gastric juice tolerance of the LAB isolate at different time intervals (0, 1, 3, and 6 h) is expressed in mean ± standard deviation with significantly different *p* ≤ 0.05 as per Duncan multiple range test and mentioned with (a, b, c, and d) superscripts.

**Figure 5:**
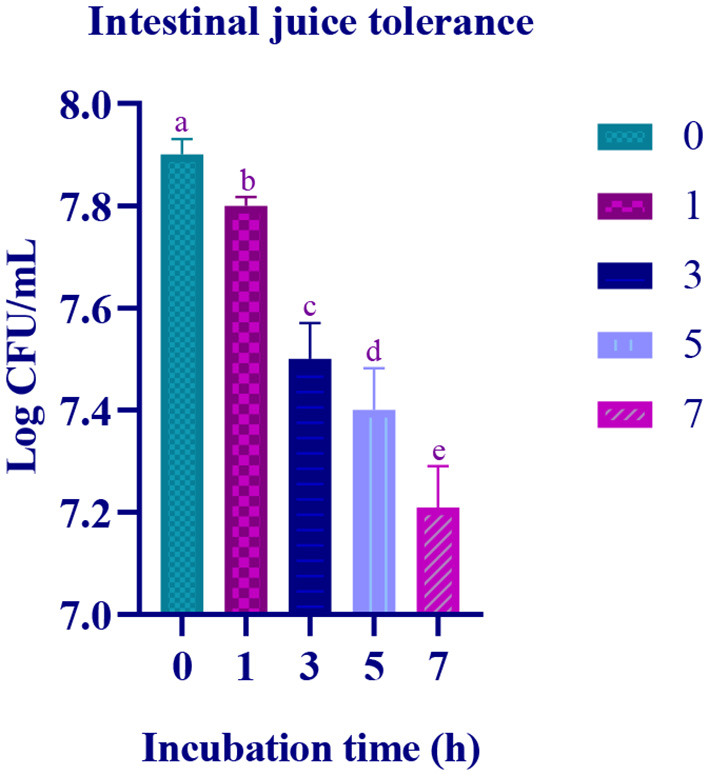
Intestinal juice tolerance of the of the LAB isolate at different time interval (0, 1, 3, 5, and 7 h) is expressed in mean ± standard deviation with significantly different *p* ≤ 0.05 as per Duncan multiple range test and mentioned with (a, b, c, d, and e) superscripts.

In the simulated gastric and intestinal juice, at the initial hour of treatment, moderate survival rate was observed and the survival rate decreased with prolonged incubation. In the last hour of the incubation, the survival rate of the isolate was 7.11 Log CFU/mL and 7.21 Log CFU/with gastric and intestinal juices, respectively (Figures 4, 5).

### Antibacterial activity

The zone of inhibition observed ranged between 6 and 18 mm. Good antibacterial (+) activity was found against *Micrococcus luteus* (MTCC 1809)*, Pseudomonas aeruginosa* (MTCC 424), and *Salmonella enterica typhimurium* (MTCC 98), and mild/moderate (∂) activity was observed against *Bacillus subtilis* (MTCC 10403)*, Escherichia coli* (MTCC 443)*, Klebsiella pneumonia* (MTCC 2822)*, Bacillus cereus* (MTCC 1272)*, Klebsiella aerogenes* (MTCC 2822), and *Pseudomonas fluorescens* (MTCC 667). No activity was observed against *Staphylococcus aureus* (MTCC 1144) (Table 3).

**Table 3:** Antibacterial activity of LAB strain isolated from fermented papaya.

**Pathogens**	**Inhibition activity**
*Micrococcus luteus* (MTCC 1809)	+
*Pseudomonas aeruginosa* (MTCC 424)	+
*Salmonella enterica typhimurium* (MTCC 98)	+
*Bacillus subtilis* (MTCC 10403)	∂
*Escherichia coli* (MTCC 443)	∂
*Staphylococcus aureus* (MTCC 1144)	∂
*Bacillus cereus* (MTCC 1272)	∂
*Klebsiella pneumonia* (MTCC 2822)	∂
*Klebsiella aerogenes* (MTCC 2822)	∂
*Pseudomonas fluorescens* (MTCC 667)	-

Symbols represent the type of inhibition: (+) good inhibition ranging from 13 to 18 mm, (∂) moderate inhibition 6 to 12 mm, and (−) no inhibition.

### Antibiotic sensitivity

Out of 13 antibiotics used, the isolate was sensitive to chloramphenicol, clindamycin, streptomycin, cefixime, gentamicin, ampicillin, tetracycline, erythromycin, rifampicin, azithromycin and resistant to kanamycin, vancomycin, and methicillin (Table 4).

**Table 4:** Antibiotic sensitivity of the isolate against different antibiotics.

**Antibiotic**	**Sensitivity**
Chloramphenicol	+
Clindamycin	+
Kanamycin	–
Vancomycin	–
Streptomycin	+
Methicillin	–
Cefixime	+
Gentamicin	+
Ampicillin	+
Tetracycline	+
Erythromycin	+
Rifampicin	+
Azithromycin	+

(+) represents sensitivity, and (–) represents resistance.

### Molecular characterization and phylogenetic analysis

**Figure 6:**
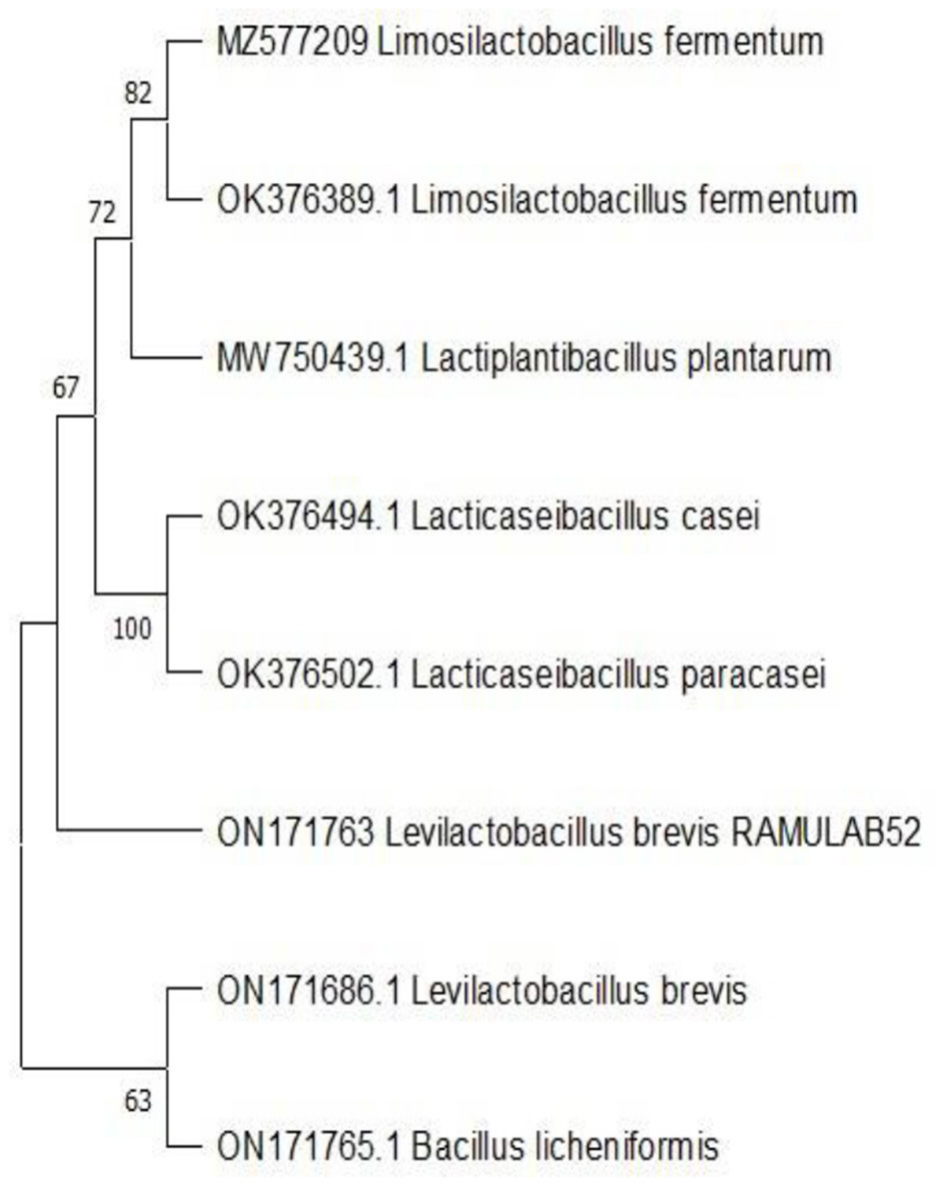
Phylogenetic tree of the LAB strain (RAMULAB52 *Levilactobacillus brevis*) isolated from fermented papaya.

The biochemical characterization and sequence of 16S rRNA region reveal the isolate as *Levilactobacillus brevis* (accession number: ON171763). The homology search of RAMULAB52 sequence had >95% similarity with *Lacticaseibacillus paracasei* and *Lacticaseibacillus casei*. Figure 6 shows the phylogenetic tree plotted using maximum likelihood.

### Adhesion to chicken crop epithelial cells, buccal epithelial cells, and HT-29 cells

Microscopic analysis reveals that the isolate was able to adhere to the chicken crop epithelial cells with a cell count of 25–40 (isolate) per cell (chicken crop epithelial cell). The isolate also had 69.33% adhesion with buccal epithelial cells and 72.92% with HT-29 cells.

### Hemolytic assay and DNase activity

The isolate used in the study is considered as γ-hemolysis as there was no formation of a lysis zone around the bacterial colonies. Similarly, no zone of inhibition was observed on performing DNase activity of the isolate, which determines that isolate used in the study showed no evidence of hemolysis or DNA degradation.

### Antioxidant activity

**Figure 7:**
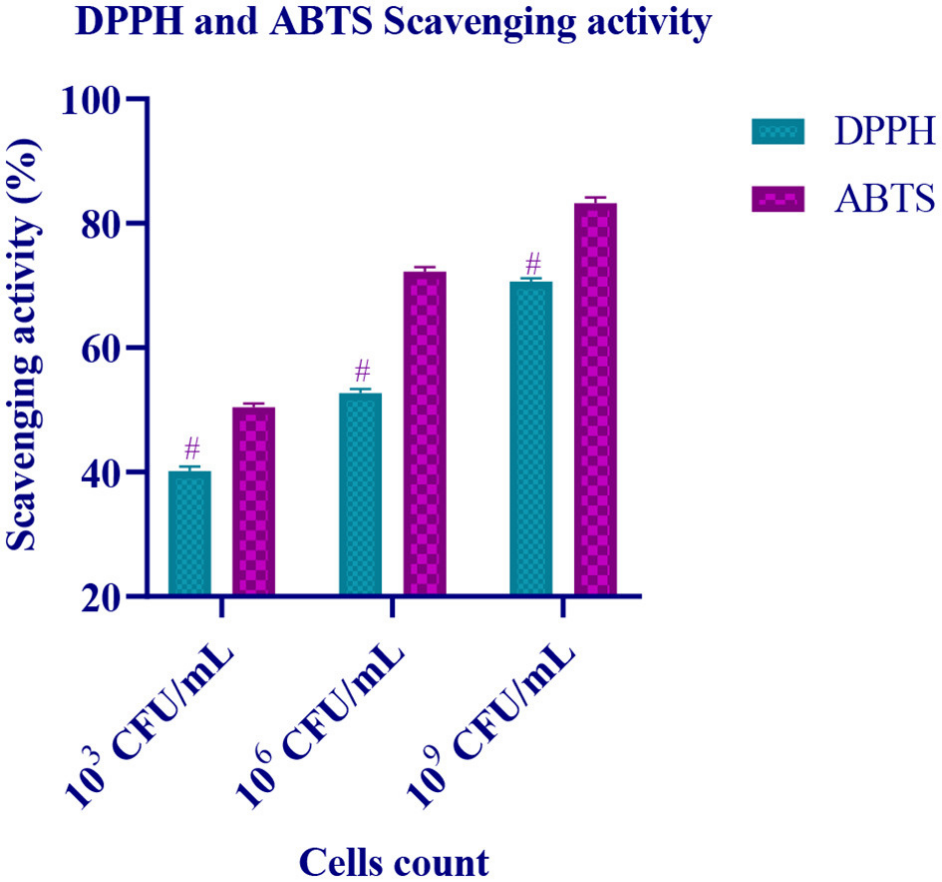
Free radical scavenging activity of different CFU/mL of LAB isolate is expressed in mean ± standard deviation with significantly different *p* ≤ 0.05 as per Duncan multiple range test and mentioned with (#) superscript.

The radical scavenging activity of the isolate (10^3^, 10^6^, and 10^9^ CFU/mL) with ABTS radicals and DPPH free radicals is represented in Figure 7. It is observed that the antioxidant activity of the isolate increases with an increase in the cell count (CFU/mL). At 10^9^ CFU/mL, the isolate has expressed high scavenging activity.

### Inhibitory activity of the enzymes α-glucosidase and α-amylase

**Figure 8:**
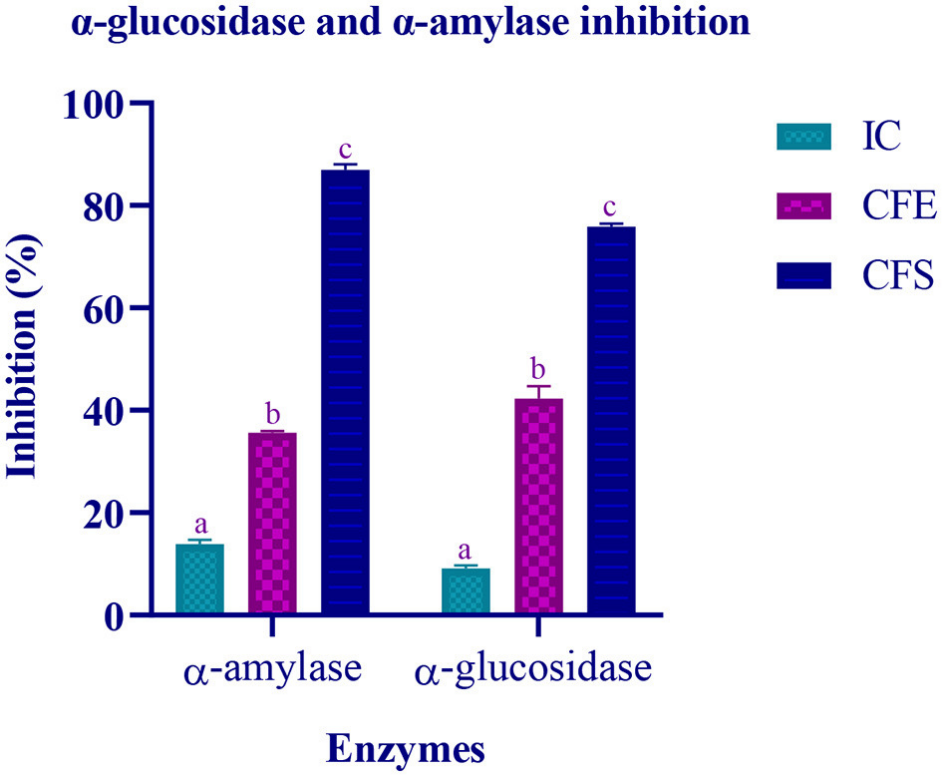
Inhibitory activity of various extracts (IC, CFE, and CFS) of LAB isolate is expressed in mean ± standard deviation with significantly different *p* ≤ 0.05 as per Duncan multiple range test and mentioned with (a, b, and c) superscript.

The inhibitory activity of the isolate against two carbohydrate hydrolyzing enzyme was performed using IC, CFE, and CFS. The target enzymes α-amylase and α-glucosidase were found to be inhibited by CFS up to 86.97% and 75.87%, respectively. Lowest inhibition was observed by IC (Figure 8).

### Determination of organic acids

The LAB strain was assessed for its production of organic acid (secondary metabolites) resulting in the following acids as represented in Table 5. The main activity of these strains is to produce end product lactic acid by metabolizing sugar in the media, but in this experiment a large amount of succinic acid (14.536 mg/mL) was also observed in comparison with the other secondary metabolites.

**Table 5:** List of organic acids extracted from LAB strains isolated from fermented papaya.

**Organic acids**	**Mean mg/mL**	**SD**
Lactic acid	1.296	0.07108
Pyruvic acid	0.995	0.082795
Malonic acid	0.276	0.011481
Maleic acid	0.050	0.001146
Fumaric acid	0.053	0.000932
Succinic acid	15.824	0.042766
Malic acid	1.014	0.037662
Tartaric acid	0.085	0.003437
Shikimic acid	0.263	0.019601
Citric acid	2.910	0.178955
Hydroxycitric acid	0.742	0.071913

### Pass pharmacological potential analysis

The Pa values obtained were greater than the Pi values for all the organic acids of LAB. The Pa activity was ranging from 0,270 to 0,708. This indicates that all the organic acids were having potential antidiabetic activity. Comparing all the obtained Pa values, tartaric acid and hydroxycitric acid have high probable activity (Table 6).

**Table 6:** Predicted PASS analysis.

**Sl. No**.	**Name of the compound**	**Activity**	**Pa**	**Pi**
1	Citric acid	Antidiabetic	0.648	0.009
2	Fumaric acid	Antidiabetic	0.512	0.021
3	Hydroxycitric acid	Antidiabetic	0.708	0.006
4	Lactic acid	Antidiabetic	0.680	0.007
5	Maleic acid	Antidiabetic	0.512	0.021
6	Malic acid	Antidiabetic	0.639	0.009
7	Malonic acid	Antidiabetic	0.270	0.100
8	Pyruvic acid	Antidiabetic symptomatic	0.228	0.095
9	Shikimic acid	Antidiabetic	0.203	0.160
10	Succinic acid	Antidiabetic	0.440	0.034
11	Tartaric acid	Antidiabetic	0.719	0.005
12	Acarbose	Antidiabetic	0.693	0.007

### Molecular docking results

The virtual screening, total number of hydrogen bonds formed, binding affinity, and intramolecular interactions are listed in Table 7. The highest docking score is observed with hydroxycitric- α-glucosidase and hydroxycitric- α-amylase complex, and it is higher than the control (acarbose) used.

**Table 7:** Virtual screening of organic acids (derivatives of LAB) against target proteins (α-glucosidase and α-amylase).

**Sl. No**.	**Name of the compound**	**Binding affinity (kcal/mol)**		**Total no. of non-bonding interactions**		**Total no. of conventional hydrogen bonds**	
		α **-glucosidase**	α**-amylase**	α **-glucosidase**	α**-amylase**	α **-glucosidase**	α**-amylase**
3	Hydroxycitric acid	−5.8	−5.5	8	4	8	4
11	Tartaric acid	−5.7	−5.5	7	4	7	4
12	Acarbose	−5.6	−5.4	7	4	6	4
1	Citric acid	−5.4	−5.4	6	5	6	5
2	Fumaric acid	−5.3	−4.2	6	2	6	2
5	Maleic acid	−5.3	−4.1	5	4	5	4
9	Shikimic acid	−5.3	−5.3	5	3	5	3
6	Malic acid	−5.2	−4.4	4	3	4	3
10	Succinic acid	−5.1	−4	3	2	3	2
7	Malonic acid	−4.8	−3.8	5	3	5	3
4	Lactic acid	−4.5	−3.6	3	4	3	3
8	Pyruvic acid	−4.5	−3.3	5	2	4	2

**Figure 9:**
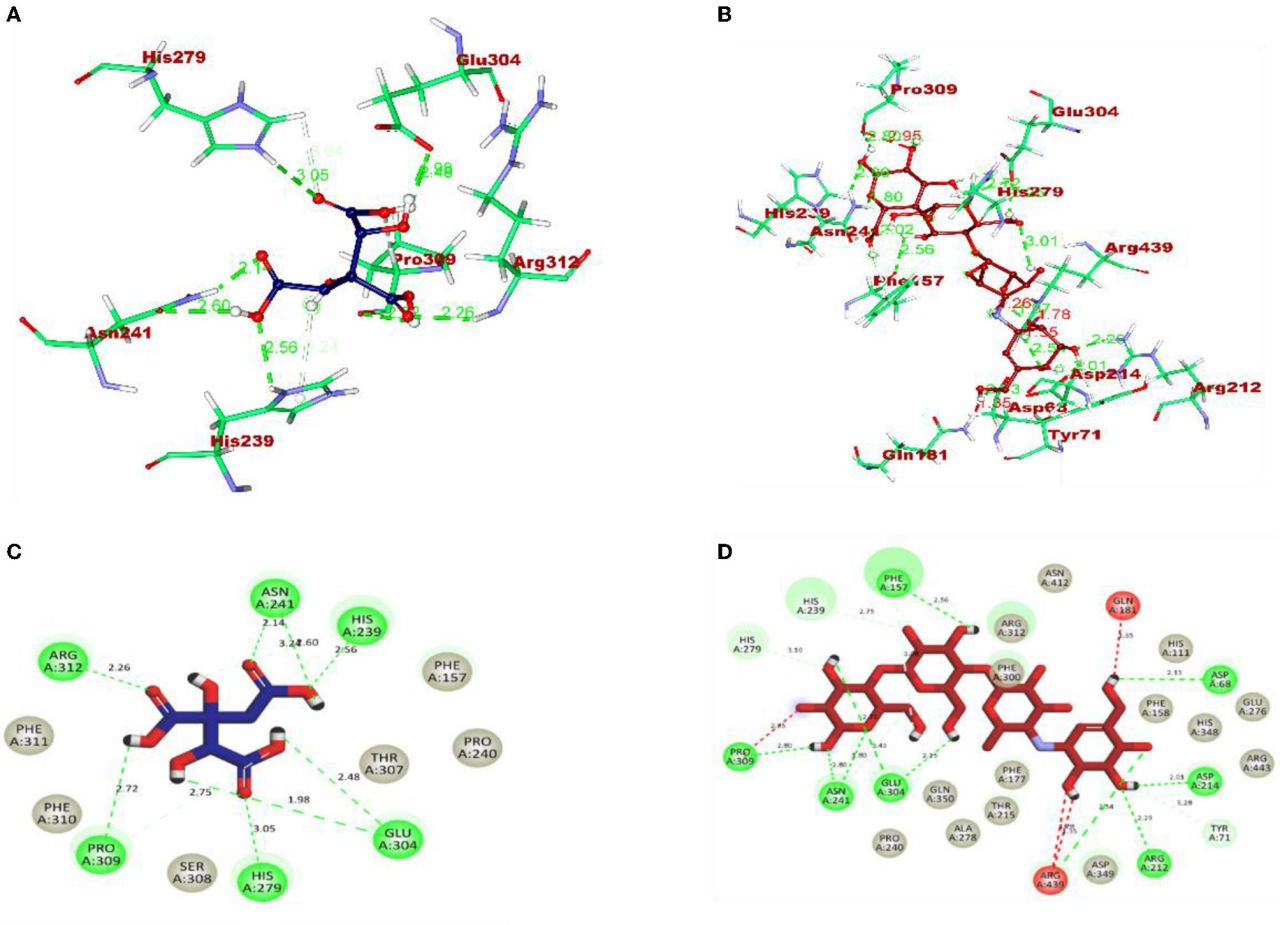
**(A)** Blue: hydroxycitric acid. **(B)** Red: acarbose. The different types of interactions and their respective distance are represented by dotted lines, and the three-letter amino acids are in marron color. 2D representation of ligands along with their bounded and non-bounded interaction of **(C)** Blue: hydroxycitric acid and **(D)** Red: acarbose with their distance is represented.

**Figure 10:**
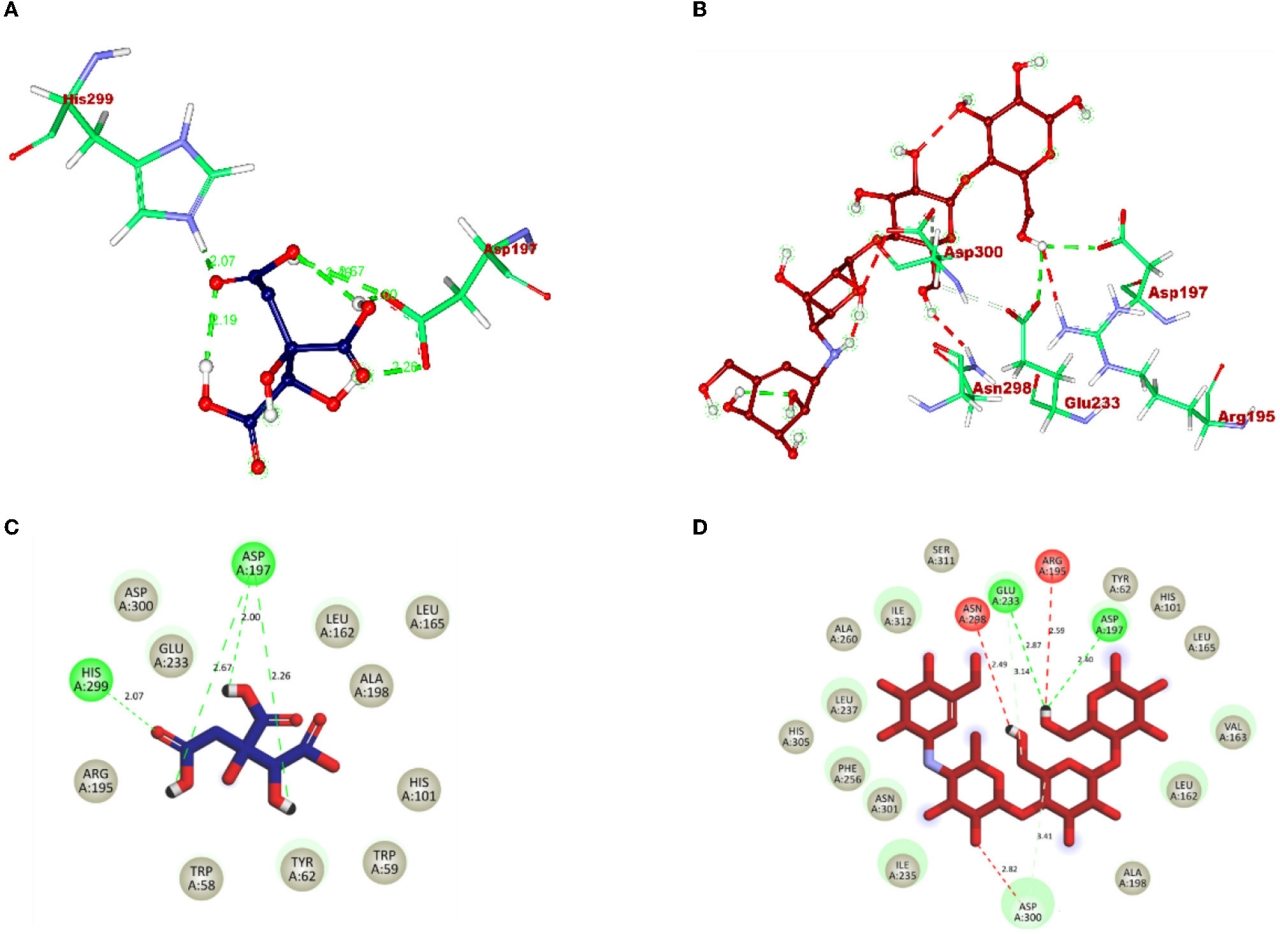
**(A)** Blue: hydroxycitric acid. **(B)** Red: acarbose. The different types of interactions and their respective distance are represented by dotted lines, and three-letter amino acids are in maroon color. 2D representation of ligands along with their bounded and non-bounded interaction of **(C)** Blue: hydroxycitric acid and **(D)** Red: acarbose with their distance is represented.

The molecular interaction of hydroxycitric acid with α-glucosidase and α-amylase had a total of eight and four non-bonded interactions, respectively. On visualizing the interaction between hydroxycitric acid with amino acid residues of α-glucosidase, it is observed that the hydroxycitric acid has interacted with ASN241, ARG312, GLU304, SER308, HIS279, PRO309, and PHE311 residues *via* hydrogen bond formation (Figure 9). Concurrently, on visualizing the interaction of hydroxycitric acid with amino acid residues of α-amylase, interaction with key residues GLU233 and ASP197 is observed (Figure 10). On comparing the obtained results with acarbose interaction with target proteins, the formed non-bonded interactions, binding affinity, and hydrogen bonds of the control with the targets are lesser than the hydroxycitric acid with target with no unfavorable acceptor–acceptor bond formation.

### Molecular dynamic (MD) simulation

**Figure 11:**
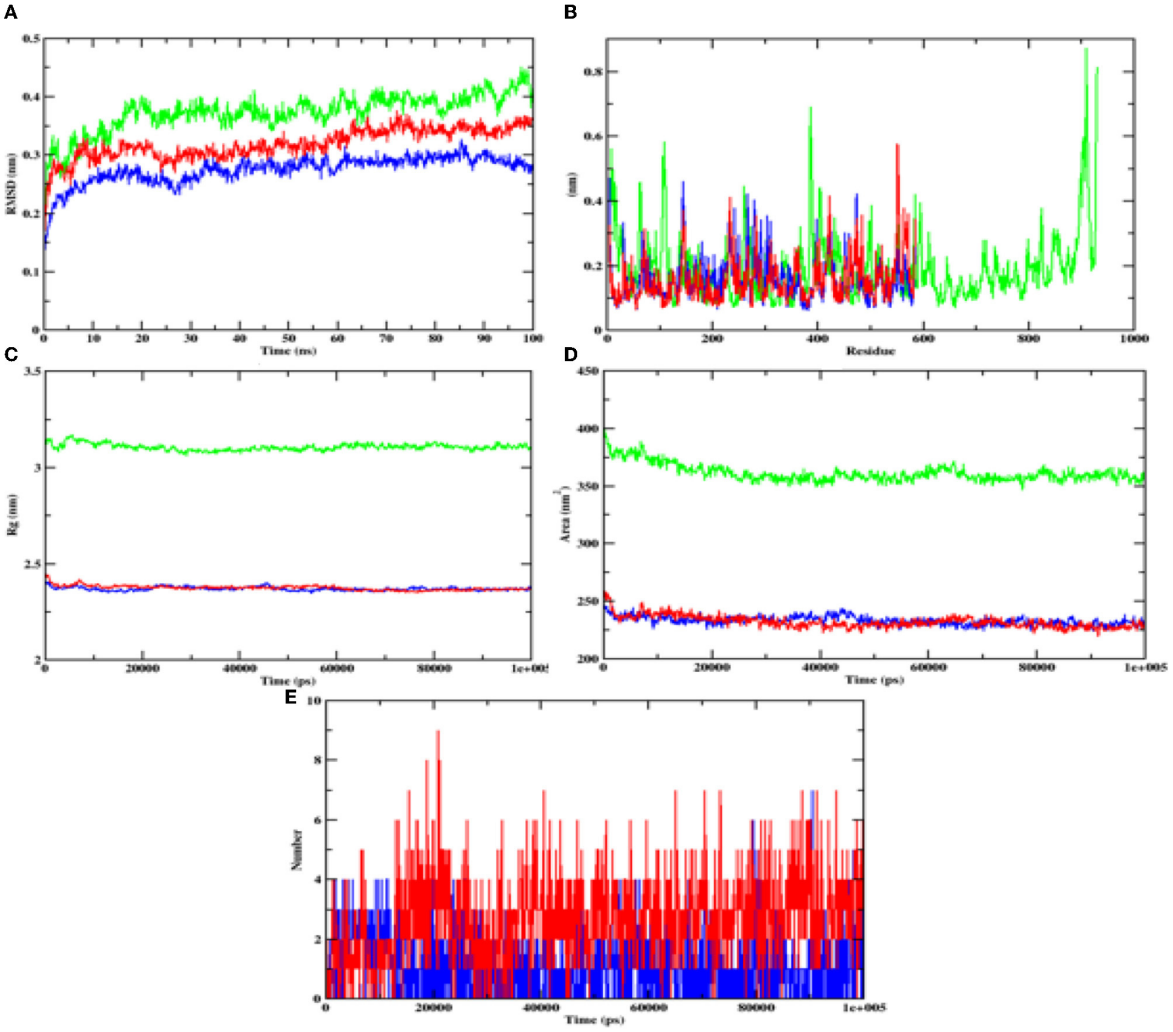
Analysis of RMSD, RMSF, Rg, SASA, and number of hydrogen bonds of hydroxycitric acid (blue) and acarbose (red) bound α-glucosidase complex and apoprotein (α-glucosidase: green) at 100 ns. **(A)** Time evolution of RMSD value of both the complexes along with protein, **(B)** RMSF, **(C)** radius of gyration (Rg), **(D)** SASA, and **(E)** hydrogen bonds.

During the MD result analysis, the RMSD graph of alpha-glucosidase depicts that both complex and apoprotein reached equilibrium after 25 ns, which interprets that the hydroxy citric acid stays inside the binding site throughout the simulation period. On comparing the RMSD value of hydroxy citric acid with acarbose, it can be said that hydroxy citric acid is more stable than acarbose, as hydroxy citric acid has reached equilibrium more rapidly. The fluctuations of hydroxycitric acid-alpha-glucosidase and acarbose-alpha-glucosidase complexes were within the RMSF range of 0.1–0.5 Å, and the RMSF plot depicts that hydroxycitric acid has lesser fluctuation compared with acarbose. Rg value of both the compound and control was found to be unchanged significantly throughout the simulation and kept fluctuating at 2.4 nm. The SASA value of both hydroxycitric acid and acarbose fluctuated within the same range of 240–250 nm. When compared with acarbose, number or H-bonds formed by hydroxycitric acid with target protein is more during 100 ns simulation (Figure 11).

**Figure 12:**
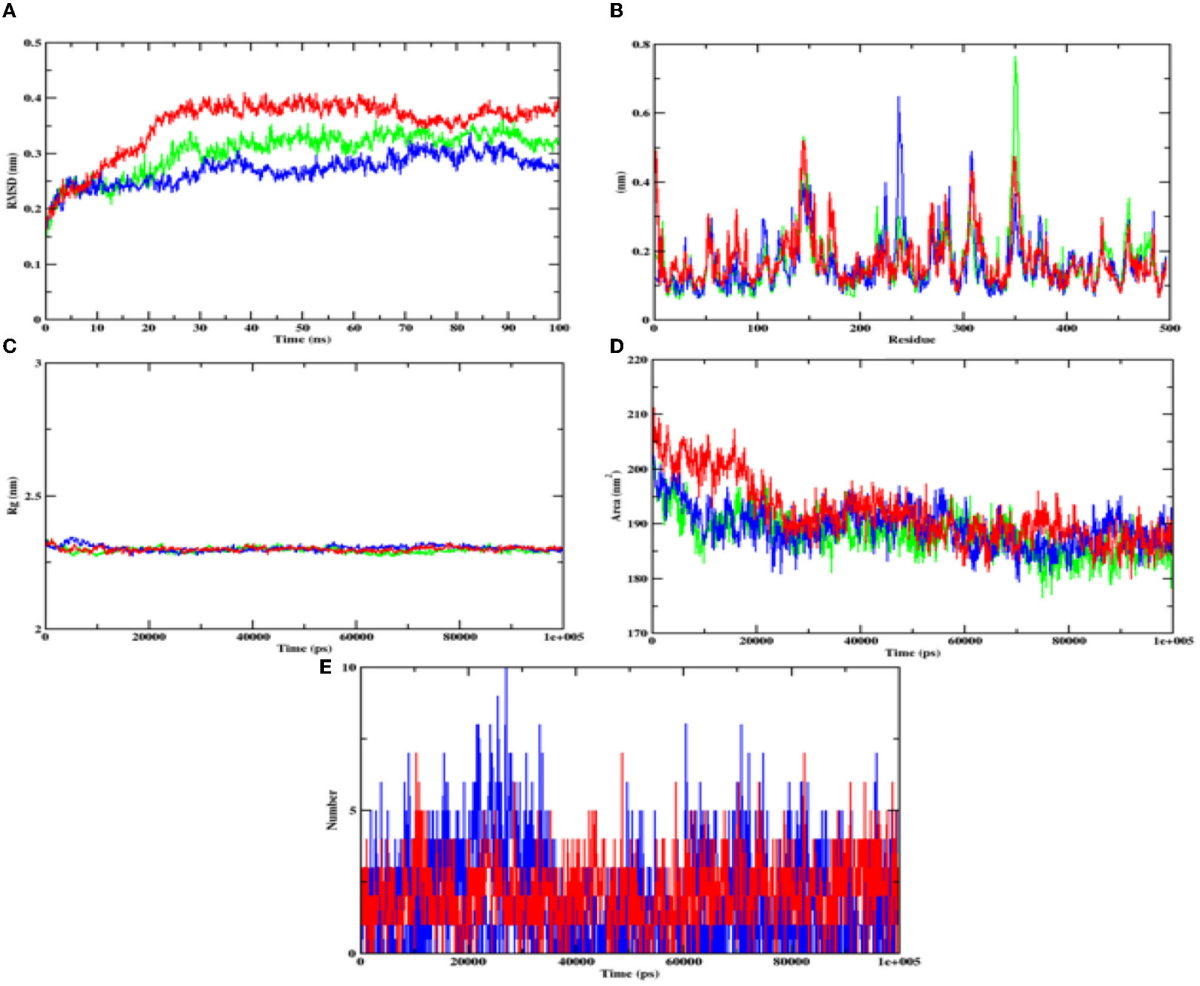
Analysis of RMSD, RMSF, Rg, SASA, and number of hydrogen bonds of hydroxycitric acid (blue) and acarbose (red) bound α-amylase complex as well as apoprotein (α-amylase: green) at 100 ns. **(A)** Time evolution of RMSD value of both the complexes along with protein, **(B)** RMSF, **(C)** radius of gyration (Rg), **(D)** SASA, and **(E)** hydrogen bonds.

In case of alpha-amylase, the RMSD value of hydroxycitric acid and apoprotein complexes was found to be in the range of 0.20–0.30 nm and the RMSD value of acarbose complex was found to be between 0.25 and 0.35 nm range. The RMSF analyses of hydroxycitric acid complex, acarbose complex, and the apoprotein were almost similar, and more or less same pattern of fluctuation was observed. The Rg value of target protein with hydroxycitric acid and acarbose complex was in the range of 2.31 nm; similarly, the SASA value of hydroxycitric acid and acarbose fluctuated within same range of 190–200 nm. Further on analyzing the formation of ligand hydrogen bond, the number of ligand hydrogen bonds formed by acarbose with target protein is lesser than the number of ligand hydrogen bonds formed by hydroxycitric acid (Figure 12).

### Binding free energy calculations

The binding free energy calculations show that hydroxycitric acid was bound with the alpha-glucosidase with higher Van der Waals energy (−220.118 kJ/mol) and binding energy (−189.1022 kJ/mol). The same pattern of results was observed in the case of alpha-amylase where Van der Waals energy (-218.568 kJ/mol) and binding energy (-180.194 kJ/mol). The different types of binding energies and their respective values of target proteins along with hydroxycitric acid and acarbose are listed in Table 8.

**Table 8:** Binding free energy values of target proteins complexed with hydroxycitric acid and acarbose.

**Protein–ligand complexes**	**Types of binding free energies**				
	**Van der Waal's energy (kj/mol)**	**Electrostatic energy (kj/mol)**	**Polar solvation energy (kj/mol)**	**SASA energy (kj/mol)**	**Binding energy (kj/mol)**
α-glucosidase-hydroxycitric acid	−220.118	−9.313	96.102	−28.166	−189.1022
α-glucosidase-acarbose	−134.192	−4.813	62.125	−9.310	−90.102
α-amylase-hydroxycitric acid	−218.568	−29.891	62.172	−21.886	−180.194
α-amylase-acarbose	−130.161	−2.106	39.340	−9.564	−87.109

## Discussion

The main objective of the present study was the isolation of probiotics that can inhibit the activity of carbohydrate hydrolyzing enzymes, α-amylase, and α-glucosidase because the present-day available treatment for diabetes using synthetic drug has been associated with after effects for a long-run consumption. Probiotics are microorganisms that tend to have health benefits when consumed as a part of the food (Syngai et al., 2015). The ability of a microorganism to survive and successfully colonize the intestine is dependent on its ability to tolerate and adapt to various environmental conditions, such as changes in temperature, pH, and salt concentrations (Divyashree et al., 2021). In this study, the isolate that was obtained has shown to have the ability to withstand and tolerate these conditions better than expected. This indicates that the isolate may have a higher chance of surviving in the intestinal environment and could potentially be used as a probiotic agent or as a functional ingredient in food products. By demonstrating better stability at varying conditions, the isolate may be considered a promising candidate for further research and development in the field of probiotics and functional foods. Based on the carbohydrate fermentation findings, the isolate is considered as hetero-fermentative in nature (Kefir et al., 2018). LAB are a well-known fact that any food consumed passes through the extreme conditions of the stomach and small intestine to reach the large intestine for absorption. Similarly, any consumed drug follows the same pattern to reach the destination (large intestine). Lactobacilli metabolize phenolics by reductases, decarboxylases, and glycosidases. Separate enzymes convert hydroxycinnamic acids and hydroxybenzoic acids. The capacity of lactobacilli to metabolize phenolics relates to their phylogeny. Metabolites of lactobacilli may contribute to the health benefits of fermented foods (Gaur and Gänzle, 2023).

The probiotics need to withstand extreme abdominal and intestinal conditions in order to exert their health beneficiary effects (Repally et al., 2017). The results in this study showed that the isolate has a remarkable ability to tolerate gastrointestinal juices. The survival rate of the isolate is on par with previous studies by Feng et al. (2017) and Guan et al. (2017) with results showing a higher potential than that showed by *Lactobacillus plantarum* (No. 14) (Nagata et al., 2009), *Lactobacillus crustorum* KH isolated from Iranian dairy products (Sharafi et al., 2014), and LAB strain isolated from poultry products and tempeh (Reuben et al., 2019; Sulistiani et al., 2020). The isolate showed a good survival rate under low pH and ox gall conditions, above 90% at the last hour of incubation at varying concentrations of ox gall, which is higher than the previously reported studies (Liong and Shah, 2005; Succi et al., 2005; Chen, 2012; Hassanzadazar et al., 2012; Li et al., 2020). It is also important to know the phenol tolerance of the isolate as microorganisms surviving inside the body release phenol, aromatic amino acids, endogenous proteins, and other toxic metabolites in the course of digestion. For a particular LAB strain, it is important to tolerate some amount of phenol. Several studies have reported on the phenol tolerance of various bacterial isolates (Gebru and Sbhatu, 2020; Andrade et al., 2022). The phenol tolerance of the LAB strains isolated from fermented beverage raabadi has ranged between 6.36 and 7.73 Log CFU/mL (Yadav et al., 2016). The viable count of isolates from fermented neera has shown a tolerance of up to 7.75 Log CFU/mL (Somashekaraiah et al., 2019). Furthermore, the *Lactobacillus sp*. G3_4_1TO2 isolated from bovine colostrum (Padmavathi et al., 2018), LAB strains isolated from fermented raw milk (Reuben et al., 2019), and rat fecal microbiota expressed the same pattern of phenol tolerance (Jena et al., 2013). In this study, the isolate has a survival rate of 70% (after 24 h incubation in 0.4% phenol), which is on par with the abovementioned studies. The obtained survival rate of the isolates helps to decide the dosage pattern during the treatment. The isolate was also studied for its ability to autoaggregate; as a result, an accumulation of cells was observed in the bottom of culture tubes. The process defines the binding of colonies belonging to the same group, which can also be called as the initial stage of biofilm formation (Trunk et al., 2018). The autoaggregation ability of the isolate in the present study is above 80%, which is much higher than the previously reported studies by Divisekera et al. (2019) and Ohn et al. (2020).

Hydrophobicity is another parameter that supports the microorganisms' adhesion to the intestinal layer. The observed hydrophobicity is directly proportional to the autoaggregation ability of the isolate. In this study, the affinity of the isolate toward the non-polar solvent xylene represents hydrophobic property. Many studies conclude that the presence of glycoproteinaceous materials on the surface of the cell wall leads to a higher hydrophobicity (Divisekera et al., 2019), which is expressed in *in vitro* conditions by the affinity of the probiotic strains toward chloroform. The isolate used in this study supports the above statement, and the results observed are on par with previous studies (Kostyukovsky et al., 2002; Ohn et al., 2020). The varying levels of coaggregation are also observed in the study, where this process helps to maintain a balanced ecosystem inside the intestine.

The antibacterial property can be considered as a forefront characteristic of any probiotic strain (Shokryazdan et al., 2014). They inhibit the pathogen growth by forming biofilm, and breaking the integrity of the cell membrane leading to an efflux of ATP which results in pore formation and growth inhibition (Shokri et al., 2017). The isolate obtained in this study has potential inhibitory activity against *Micrococcus luteus* (MTCC 1809), *Pseudomonas aeruginosa* (MTCC 424), and *Salmonella enterica typhimurium* (MTCC 98). This isolate was also assessed for antibiotic sensitivity, and it is observed that the isolate is sensitive to chloramphenicol, clindamycin, streptomycin, cefixime, gentamicin, ampicillin, tetracycline, erythromycin, and rifampicin. This assessment will help the patients who are sensitive to probiotics, i.e., there are possibilities that cause probiotic consumers develop allergic reactions, and consumption of multiple probiotics can make the immune system exhibit hypersensitive reactions. To address these side effects, the antibiotic sensitivity of the probiotics is highly important (Hill et al., 2014). The results reveal that the LAB isolate from the present study is resistant to kanamycin, vancomycin, and methicillin, which also serve the consumer's requirement and give a space for probiotics and antibiotic combined therapies in conditions, such as female (UTI) urogenital tract infection, infective endocarditis, and diarrhea.

Furthermore, it is also equally important to know the adhesion ability of the isolate with the epithelial cell of the intestine. The ability of probiotic bacteria to adhere to the epithelial cells of the intestine is crucial for colonization, modulation of the immune response, and delivery of the probiotics to the target site (González-Rodríguez et al., 2012). It helps to establish the probiotic bacteria in the gut, interact with host cells, and improve gut barrier function. Therefore, the adhesion ability of probiotics to the intestinal epithelium is an essential factor for their beneficial effects on the host (Liu et al., 2010). The isolate has expressed a good adhesion with chicken crop epithelial cells, buccal epithelial cells, and HT-29 cells. DNase hydrolysis assay was performed to determine the pathogenicity of the isolate and DNase enzyme activity. As the isolate lacks DNase, no cell death was observed, no hemolysis was observed in the hemolytic assay, and similar results are expressed by probiotic strains LMEM6, LMEM7, and LMEN8 isolated from curd sample (Halder et al., 2017). These two properties of the isolate indicate the potential and safety of the isolate. It is hypothesized that the antioxidants consumed in the diet have a potential effect on diabetes by inhibiting reactions of peroxidation. The isolate used in the investigation for DPPH and ABTS scavenging activity showed good scavenging activity, and it is on par with *L. plantarum* AR113 and *L. paracasei* (isolated from fermented yak milk) (Lin et al., 2018; Li et al., 2022). ABTS scavenging activity of the isolate was found to be higher than *Lactilactobacillus curvatus* MG5020*, Lactilactobacillus sankei* MG5048 (Kim et al., 2022)*, and Lactobacillus brevis* KU15151 (Yang et al., 2020). The main aim of the study was to know the ability of the isolate to inhibit the carbohydrate hydrolyzing enzymes (α-glucosidase and α-amylase). The inhibitory activity of the isolate was found to be 86.97% and 75.87% for α-glucosidase and α-amylase, respectively, with CFS. It is a well-known fact that the enzyme α-glucosidase present in the border of the small intestine, takes part in the cleavage of oligosaccharides and starch into monosaccharides, activity of this enzyme leads to increased blood glucose levels (Hanefeld, 2018). The commercially available acarbose is used in an effective management of the enzyme α-glucosidase (Wihansah et al., 2018). Based on the results obtained, it can be predicted that the isolate obtained can act as an effective inhibitor of α-glucosidase. The observed inhibition was much greater than the inhibitory effect on α-glucosidase caused by the LAB strains isolated from Indonesian kefir grains (Yusuf et al., 2021), MBEL1397 and KU15006 isolated from kimchi (Son et al., 2017), and MBEL1361 isolated from Korean traditional fermented food (Kwun et al., 2020). In comparison with LAB isolated from quinoa-based fermented yogurt (Ujiroghene et al., 2019) and *Lacticaseibacillus casei* (RAMULAB07 and RAMULAB08) and *Limosilactobacillus fermentum* (RAMULAB09, RAMULAB10, RAMULAB11, and RAMULAB12) isolated from dosa batter (Kumari et al., 2022). In addition, the isolate used in the present study showed a higher ability to inhibit α-amylase compared to *L. plantarum* DMR15 (Tirwa et al., 2020) and camel milks fermented by *L. plantarum* DSM2468, *L. reuteri*-KX88apoprotein1777, and *L. plantarum*-KX881779 (Ayyash et al., 2018). In this study CFS exhibited the best inhibiition against α-glucosidase and α-amylase (carbohydrate hydrolyzing enzymes) in comparison to IC and CFE. Based on the obtained values from PASS analysis, it can be predicted that the organic acids from LAB have potential antidiabetic activity as the Pa values of all the organic acids are higher than Pi values. On visualizing the interaction between hydroxycitric acid with amino acid residues of α-glucosidase, it is observed that the hydroxycitric acid has interacted with ASN241, ARG312, GLU304, SER308, HIS279, PRO309, and PHE311 residues *via* hydrogen bond formation and the observed interactions are on par with the previous studies (Prabhakaran et al., 2022; Shivanna et al., 2022); the commercial acarbose which is widely used for its antidiabetic activity has a similar interaction with the target protein. Concurrently, on visualizing the interaction of hydroxycitric acid with amino acid residues of α-amylase, interaction with key residues GLU233 and ASP197 is observed. Using docking results, the possible interaction between hydroxycitric acid and target proteins (complex) has been understood. The overall outcomes of the MD studies indicate the conformational stability of hydroxycitric acid in binding to the target protein. The outcomes also suggest that the binding site had less influence on the structures. The ligand hydrogen bond analysis during MD simulation also indicates that hydroxycitric acid–alpha-glucosidase complex is more stable than acarbose–alpha-glucosidase complex. In case of both alpha-glucosidase and alpha-amylase proteins, the protein-ligand complexes formed with hydroxycitric acid were found to be stable comparatively with those of acarbose, based on the obtained values and observed fluctuation. The MM/PBSA is the most used approach for calculating binding free energies. The study of binding free energy determines van der Waal's energy and binding energies have an important impact in developing protein–ligand complexes during MD simulation. Our results in MM/PBSA show that hydroxycitric acid is the most favored binding ligand. With respect to the Pa values, molecular docking results, MD results, and binding free energy calculations, hydroxycitric acid has been considered as the most potent inhibitor of both the carbohydrate digestive enzymes. Even the molecule has shown its antidiabetic potential in biological studies, which is similar to a recent study, where butanedioic, lactic, and gallic acid were found with the same antidiabetic (α-amylase and α-glycosidase inhibition) potential (Wen et al., 2023). Therefore, our study reports hydroxycitric acid as the potential antidiabetic agent with the potential to inhibit both the carbohydrate digestive enzymes. In the near future, we are aiming to conduct *in vivo* studies using the same.

## Conclusion

This study presents a novel approach to isolate potential probiotic LAB from fermented papaya with antidiabetic properties. The results demonstrate that the isolated strain is safe for consumption in food for two reasons: first, it is generally recognized as safe (GRAS) and isolated from food sources and secondly, no hemolysis or DNA degradation was found. Additionally, the isolate was able to tolerate acid bile, phenol, and gastrointestinal conditions. Additionally, the isolate displayed autoaggregation ability, hydrophobicity, and antibiotic sensitivity and exhibited antimicrobial activity against pathogenic bacteria. The *in vitro* and *in silico* data provided in the study strongly support the inhibitory activity of this probiotic against the enzymes α-glucosidase and α-amylase, which are known to contribute to the development of type 2 diabetes. The outcomes suggest that hydroxycitric acid could be the most potent organic acid responsible for this antidiabetic activity, resulting in the effective inhibition of both the carbohydrate digestive enzymes. In the coming research, our primary attention will be directed toward carrying out *in vivo* investigations to evaluate the effectiveness and safety of the strain for potential utilization as an antidiabetic component. Overall, the findings suggest that the probiotic bacteria isolated from fermented papaya could be recommended as a potential antidiabetic component in food products. Furthermore, these results could pave the way for the isolation of even more potent probiotic strains from fermented fruits.

## Data availability statement

The datasets presented in this study can be found in online repositories. The names of the repository/repositories and accession number(s) can be found in the article/supplementary material.

## Author contributions

RR designed the research study. NS, VK, and SH performed the research activities. MJ analyzed the data and validated the study. A-BA-O and VL wrote the manuscript and edited the manuscript submitted. All authors have given their approval for publication.
